# Supplementary material for: Giant magnetoresistance, three-dimensional Fermi surface and origin of resistivity plateau in YSb semimetal
Source: Sci Rep. 2016 Dec 9;6:38691. doi: 10.1038/srep38691 (PMC5146676; doi:10.1038/srep38691)
Supplement: Supplementary Materials [file srep38691-s1.pdf]

SUPPLEMENTARY MATERIAL

Giant magnetoresistance, three-dimensional Fermi surface and origin of resistivity plateau in YSb semimetal

Orest Pavlosiuk, Przemysław Swatek and Piotr Wiśniewski

Institute of Low Temperatures and Structure Research, Polish Academy of Sciences, Wrocław, Poland

1 Resistivity and its universal low-temperature plateau

In Fig. S1(a) we show metallic-like temperature dependence of electrical resistivity ρ of samples #1 and #2. The room- to residual-resistivity ratio, RRR $(\equiv \rho(300\,\mathrm{K})/\rho(2\,\mathrm{K}))$, is 13 and 22, respectively. Behavior of $\rho(T)$ of sample #1 in different applied magnetic fields is shown in Fig. 1(b). Plateau at $T \leq 15$ K is observed independent of strength of applied field.

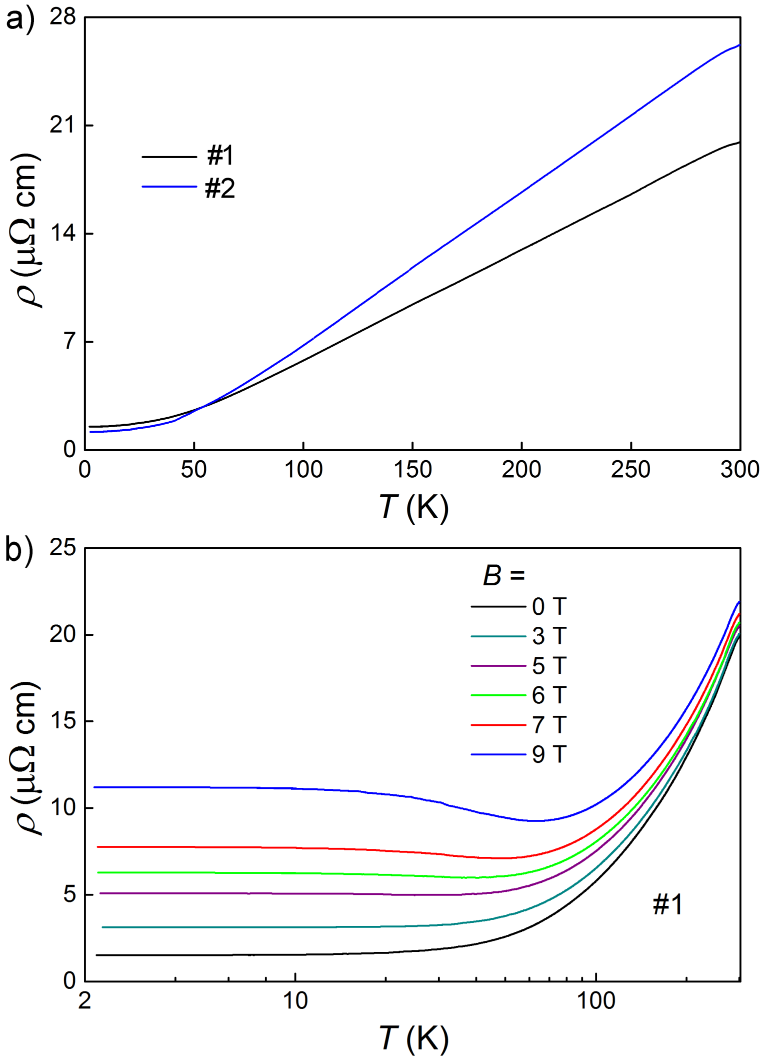

Figure S1. (a) Resistivity of YSb versus temperature for samples #1 and #2. (b) Resistivity of sample #1 versus temperature, measured in different magnetic fields.

2 Determination of effective masses

Observation of clear SdH oscillations at different temperatures allowed us to determine effective masses of charge carriers responsible for their strongest components. Fitting of FTT amplitudes for components denoted as α and β with $R_{i(=\alpha,\beta)}(T) = (\lambda m_i^* T/B)/\sinh(p\lambda m_i^* T/B)$ function with B set at 9 T and constant $\lambda = 2\pi^2 k_B m_0/e\hbar$ ($\approx$ 14.7 T/K), as shown in Fig. S2, yielded effective masses: $m_\alpha^* = 0.29m_0$ and $m_\beta^* = 0.3m_0$. In their recent arXiv preprint Yu et al. reported $m_\alpha^* = 0.17m_0$ and $m_\beta^* = 0.27m_0$.[1]

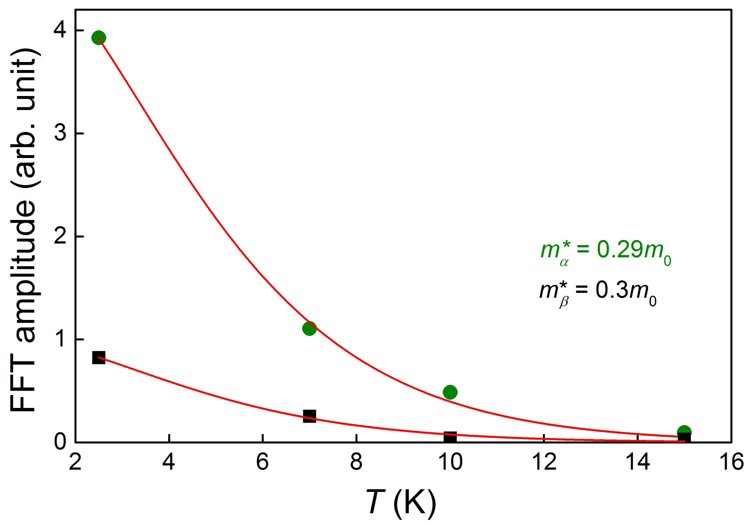

Figure S2. Amplitudes of SdH oscillations corresponding to orbits α (circles) and β (squares) obtained from FFT analysis. Red lines represent fits with function described in text yielding $m_\alpha^* = 0.29m_0$ and $m_\beta^* = 0.3m_0$.

3 Multi-component Lifshitz-Kosevich function

The oscillatory component of resistivity in magnetic field may be expressed as:[2–4]

$$\Delta\rho_{xx} = {}^5/_2 \sum_i \sqrt{\frac{B}{2p_i f_i}} R_{T,i} R_{D,i} R_{S,i} \cos\left(2\pi\left(p_i f_i/B - p_i\varphi_i - \frac{1}{8}\right)\right) =$$

$$= {}^5/_2 \sum_i \sqrt{\frac{p_i}{2f_i B}} \frac{\lambda m_i^* T \, \exp(-p_i\lambda m_i^* T_{D,i}/B)\cos(p_i\pi m_i^* g_i^*)}{\sinh(p_i\lambda m_i^* T/B)} \cos\left(2\pi\left(p_i f_i/B - p_i\varphi_i - \frac{1}{8}\right)\right), \qquad (S1)$$

where for i-th SdH component:

f_i is frequency,
$R_{D,i} = \exp(-p_i\lambda m_i^* T_{D,i}/B)$ – the Dingle reduction factor,
$R_{T,i} = (p_i\lambda m_i^* T/B)/\sinh(p_i\lambda m_i^* T/B)$ – the temperature reduction factor and
$R_{S,i} = \cos(p_i\pi m_i^* g_i^*)$ – the spin factor.

φ_i is the phase, p_i denotes harmonic number , m_i^* cyclotron mass (in units of free electron mass m_0), g_i^* effective g-factor and $T_{D,i}$ the Dingle temperature. $\lambda = 2\pi^2 k_B m_0/e\hbar$ ($\approx$ 14.7 T/K) is constant.

In order to fit complex $\Delta\rho_{xx}$ data shown in Fig. 6 without preassuming the harmonic numbers of components we used a simplified version of the Equation S1:

$$\Delta\rho_{xx} = \sum_i a_i \sqrt{1/B} \frac{\exp(-c_i/B)}{\sinh(b_i/B)} \cos\left(2\pi\left(f_i/B - \varphi_i - \frac{1}{8}\right)\right). \qquad (S2)$$

Here $\exp(-c_i/B)$ represents $R_{D,i}$, whereas $a_i\sqrt{1/B}/\sinh(b_i/B)$ $[\propto \sqrt{B}R_{T,i}R_{S,i}]$ comprises $R_{T,i}$ and $R_{S,i}$ (with $b_i = p_i\lambda m_i^*T$ and $c_i = p_i\lambda m_i^*T_{D,i}$).
Initial fit revealed that $\varphi_2 = 2\varphi_1$ and $\varphi_4 \approx 3\varphi_1$, thus we could identify second and fourth component as second and third harmonic, respectively, of the strongest oscillation with $\varphi_1 = 360\,\mathrm{T}$.
Accordingly, we put constraints on parameters: $b_2 = 2b_1$, $b_3 = 3b_1$, $c_2 = 2c_1$ and $c_3 = 3c_1$.

For $T = 2.5\,\mathrm{K}$ (temperature of our measurement), $m_1^* = 0.29$ and $m_3^* = 0.3$ (since $m_\alpha^* = 0.29m_0$ and $m_\beta^* = 0.3m_0$, as it was shown above) one obtains $b_1 = 11.51\,\mathrm{T}$ and $b_3 = 11.9\,\mathrm{T}$.
When b_1 and b_2 were fixed at these values, the final fit yielded parameters collected in the Table below:

Supplementary Table
Parameters obtained from fit of the multi-component Lifshitz-Kosevich function to data-points shown in Fig. 6 of the main text. Effective masses and Dingle temperatures calculated from b_i and c_i parameters are also shown.

$i =$	1	2	3	4	5	6
f_i (T)	360	720	740	1072	1160	1430
FS-sheet assignment:	α	2α	β	3α	δ	α_1
φ_i	0.74(3)	0.50(4)	0.74(2)	0.60(2)	0.68(3)	0.31(2)
a_i	0.41	0.41	0.11	0.41	0.005	0.0037
b_i	11.51	23.02	11.91	34.53	30	40
m_i^* (m_0)	0.29	–	0.3	–	0.76	1.00
c_i	4.56	9.11	22.93	13.67	54.24	64.00
$T_{D,i}$ (K)	1.07	–	5.2	–	4.9	4.35

The ratio $m_6^*/m_1^*(= m_{\alpha_1}^*/m_\alpha^*) = 3.48$ is the anisotropy of effective mass of electrons on the α-sheet of FS. This value is in excellent agreement with values of 3.4 and 3.6 derived from angular behavior of MR and SdH oscillations, respectively.

References

1. Yu, Q.-H., Wang, Y.-Y., Xu, S. & Xia, T.-L. Magnetoresistance and Shubnikov-de Hass oscillation in YSb. *arXiv* (2016). URL http://arxiv.org/abs/1604.05912. 1604.05912.

2. Lifshitz, I. M. & Kosevich, L. M. On the Theory of the Shubnikov-De Haas Effect. *Sov. Phys. JETP* **6**, 67–77 (1958). URL http://www.jetp.ac.ru/cgi-bin/e/index/e/6/1/p67?a=list.

3. Shoenberg, D. *Magnetic Oscillations in Metals* (Cambridge University Press, Cambridge, 1984). URL http://dx.doi.org/10.1017/CBO9780511897870.

4. Seiler, D. G. The Shubnikov-de Haas Effect: A Powerful Tool for Characterizing Semiconductors. In Landwehr, G. (ed.) *High Magn. Fields Semicond. Phys. II*, 578–587 (Springer, 1989). URL http://link.springer.com/10.1007/978-3-642-83810-1_90.
